# Does antenatal care service quality influence essential newborn care (ENC) practices? In Bahir Dar City Administration, North West Ethiopia: a prospective follow up study

**DOI:** 10.1186/s13052-018-0544-3

**Published:** 2018-08-29

**Authors:** Tadese Ejigu Tafere, Mesganaw Fanthahun Afework, Alemayehu Worku Yalew

**Affiliations:** 1 School of Public Health (SPH), College of Health Sciences, Addis Ababa University, Addis Ababa, Ethiopia; 2 School of Public Health, College of Medicine and Health Sciences, Bahir Dar University, Bahir Dar, Ethiopia

**Keywords:** Newborn care, Cord care, Thermal care, Neonatal mortality

## Abstract

**Background:**

The neonatal period is only 1/60th of the first 5 years of life but it accounts for 63% of all infant deaths and 44% of all under-five deaths in Ethiopia**.** Most causes of neonatal death are preventable with clean cord care, temperature control by delaying first bath and initiation of early breastfeeding which has additional benefit of controlling hypothermia. Poor positive pressure ventilation (PPV) with ambubag is also another essential neborn care practice to reduce neonatal death even though it was not the focus of this study with the assumption that it cannot be measured only by exit interview (needs direct observation about the procedure).

This study was aimed to assess the link between quality of ANC service and implementation of essential newborn care practices among pregnant women attending ANC at public health facilities of BDR City Administration.

**Methods:**

A facility based prospective follow up study was conducted and 970 pregnant women with gestational age ≤ 16 weeks who came for their first ANC visit were enrolled. Women were followed from their first ANC visit until 6 weeks after delivery. Longitudinal data was collected during consultation with ANC providers using structured observation checklist and exit interview was also carried out at 6 weeks after birth when they came to immunize their child to assess the essential newborn care practices that their babies received. ANC service was considered as acceptable quality if women received ≥75th percentile of the essential ANC services. Generalized Estimating Equation (GEE) was carried out to control cluster effect among women who received ANC in the same facility.

**Results:**

The composite essential newborn care practice indices were 13.7%, with 95% CI (11.3%, 16.2%) and 86.3%, with 95% CI (83.8%, 88.7%) for good and poor essential new born care practices respectively. Of those who received acceptable ANC quality and un acceptable ANC quality 24.7% and 9.6% had good essential newborn care practice respectively (X^2^ = 31.668, *p* < 0.000).

**Conclusions:**

Most neonatal interventions are not reaching newborns, indicating a “policy-to practice gap”. It is crucial that maternal knowledge about essential newborn care need to start before the baby’s birth with an effective educational plan.

Quality ANC service is a facilitator for essential newborn care practice. To improve newborn survival, newborn care should be integrated into the current maternal and child health interventions, and should be promoted both at community and health facility level as part of a universal coverage strategy.

**Electronic supplementary material:**

The online version of this article (10.1186/s13052-018-0544-3) contains supplementary material, which is available to authorized users.

## Background

It is estimated that each year 4 million neonatal deaths occur globally and almost exclusively (99%) in low income countries [1].

In developing countries, progress towards achieving Millennium Development Goal 4 - to reduce under-5 mortality by two-thirds from the 1990 baseline is being hampered by slow progress in reducing neonatal death. Neonatal mortality has remained resistant to change [2–4].

Most causes of neonatal death are preventable and related to cord care to decrease sepsis, temperature control by delaying first bath and initiation of early breastfeeding which has the additional benefit of controlling hypothermia [5].

The neonatal period is only 1/60th of the first 5 years of life but it accounts for 63% of all infant deaths and 44% of all under-five deaths in Ethiopia. Between 2000 and 2016, under-five mortality in Ethiopia declined dramatically, from 166 to 67 deaths per 1000 live births. Nevertheless, like other developing countries, the reduction is mainly a result of fewer deaths in children one to 59 months old, while neonatal (first 28 days of life) mortality has shown little change (dropping from 49 to 29 deaths per 1000 live births) [6, 7].

The World Health Organization recommends improving care practices at birth in order to reduce neonatal morbidity and mortality. These have been described as essential newborn care (ENC) practices and include clean cord care, thermal care and initiating breast feeding immediately or within the first hour after birth [8].

One of the instruments to obtain reduction in neonatal mortality is antenatal Care (ANC) by counselling pregnant women to have access for essential newborn care practices during delivery at health facility and preparing them to care for their newborn [9].

The period following birth is often marked by cultural practices in Sub-Saharan Africa (SSA). Some cultural practices hinder the health and survival of the newborn, like bathing the baby immediately after birth, putting different substances on the umbilical cord and giving different prelacteal feeds for the newborn. Even though understanding these cultural practices is an important part of ensuring effective and timely newborn care; there is limited documentation of this evidence in SSA where suboptimal newborn care practices has been widely reported [10].

Therefore the aim of this study was to assess whether or not quality antenatal care lead to improve essential newborn care practices which might have an input for the design of programs and policies to improve child survival in developing countries like Ethiopia.

## Methods

### Study design, setting and population

A facility based prospective follow up study was conducted from October 2015 to August 2016 in Bahir Dar City Administration, Amhara Regional State, which is located in the North West part of Ethiopia.

According to the Amhara National Regional State Bureau of Finance and Economic Development report, the projected population by 2015/16 was 297,775 (80.5% urban Vs 19.5% rural), of these 156,515 (52.6%) were females & there were 3300 eligible pregnant women.

The study was conducted on seven public health facilities, one hospital and six health centers. Currently two public hospitals and ten public health centers are providing maternal health care services but at the beginning of the data collection period of this study, one of the hospitals and two of the health centers were new and they were not included in the studied health facilities due to less flow of ANC service users.

All selected first visit pregnant women with gestational age ≤ 16weekss attending ANC during data collection period were included in the study with voluntary participation. Those women with pregnancy outcome of intrauterine fetal death and a still birth baby were excluded in the analysis as data on newborn care practices were not applicable.

### Sample size and sampling technique

The current study is part of a large follow up study with multiple objectives. The detail of the sample size calculation assumptions to address all objectives is described in the other part of the study published on Plos ONE [11]. For the current study a sample size was calculated with the prevalence of optimal cord care (24.0%) among women with ≥4ANc visits [10] considering these women as if they received acceptable quality ANC service due to the absence of other literature that showed ANC quality versus essential newborn care practice. In addition we assume that there could be a 10% reduction in optimal cord care among mothers who received poor quality ANC service and 80% power,95% confidence level, ratio of un exposed (women who received unacceptable quality ANC service) to exposed (women who received acceptable quality ANC service) is 2:1and 15% non response rate. The final calculated sample size was 656. The optimal thermal care and breastfeeding care were also considered to calculate the sample size but the sample size calculated using the optimal cord care was the maximum. However, to increase the power of the study, all 823 women who gave birth alive baby were included in the analysis.

### Data collection procedure

Seven female diploma Midwives and two Bachelor of Science female Midwives who were not the staffs in the study health facilities were recruited as data collectors and supervisors respectively to observe the quality of ANC service provided to pregnant women during their ANC visits using a structured observation check list adapted from the focused antenatal care protocol [12]. In addition, there was also an exit interview with women about their essential newborn care practices while they came to health facilities for immunization of their child. If women do not came to health facilities for immunizing their child, the data collectors were tracing them at home, based on their address registered during the first visit.

*Dependent****-*** Essential newborn care practice (good/poor) which is a composite variable that includes clean cord care, optimal thermal care and good neonatal feeding practices. Poor positive pressure ventilation (PPV) with ambubag is also another essential newborn care practice to reduce neonatal death even though it was not part of this study that might be our assignment in the next time.

Good newborn care practice (if all the three of them are full filled) was coded as “1” otherwise “0” that indicates poor practice

- *▪ Clean cord care:* use of a clean cutting instrument to cut the umbilical cord plus clean thread to tie the cord plus no substance applied to the cord.
- *▪ Optimal thermal care:* baby wrapped at birth plus first bath after 24 h.
- *▪ Good neonatal feeding****:*** initiating breastfeeding within the first 1 h after birth plus baby given no supplements at all in the first month of life.

*Independent*- quality of ANC service was the primary exposure of interest. The other exposure variables include: place of delivery, parity, residence, educational status, maternal occupation, marital status, maternal age,

Quality of ANC service is a composite index which was assessed using structured pre-defined observation check list of 88 items against the standard for each visit that is developed on the basis of the Ethiopian Federal Ministry of Health management protocol on selected obstetric topics [12]. The tool is validated in Ethiopian context by Federal Ministry of Health. The components of ANC services assessed were categorized into three groups: (i) Comprehensive history taking (36 items) (ii); Measurements, tests and treatments (29 items) and (iii) provision of information (23 items). Data collection tool for the larger study is attached as Additional file 1.

If the essential ANC services were given it was coded as 1 otherwise 0. Overall quality score and percentile was computed to categorize the quality of the services a woman received. If she scored ≥75th percentile, of essential ANC services, ANC service was considered acceptable quality otherwise not [13].Calculation of a composite index for the overall ANC service quality received by each woman is described in detail elsewhere.

Seven female diploma midwife data collectors and two Bachelor of Science in Midwifery supervisors experienced in data collection were involved in the data collection and supervision respectively. Both the data collectors and supervisors were not working in the study health facilities. Two days training about the data collection instrument going through question by question; about how to observe ANC service provision supported with practical exercises (role plays) and how to conduct exit interview to assess essential newborn care practice was given for both data collectors and supervisors.

Data Analysis**:** Data were coded and entered into EPI data version3.1 and exported to SPSS version 20 for analysis. Descriptive statistics were used to describe the data. Generalized estimating equation analysis with binary response variable using robust estimator and exchangeable working correlation matrix was carried out to control the cluster effect of the data among women who received ANC services within the same facility by the same ANC provider. Based on Hosmer and Lemeshow applied logistic regression guide a *p*-value < 0.2 was considered to select eligible variables for the final model and *p*-value < 0.05 was considered to identify statistically significant predictor variables for essential newborn care practice.

## Results

### Background characteristics of respondents

Among 970 enrolled mothers 823 (84.8%) completed the follow up (from their first ANC visit to 6 weeks post partum period).However, there was no statistically significant difference in background characteristics and quality of ANC services received between those who had loss to follow up and those who completed the follow up (quality of ANC service for those loss to follow up was considered by the services received only in the first visit). The mean age of respondents was 25.46 ± 4.37 years; majorities (88.5%) were from urban and 21.0% of them had no formal education (Table 1).

**Table 1:** Socio-demographic characteristics of study participants in public health facilities of Bahir Dar City administration (*N* = 823), October 2015 to August 2016

Variables	Frequency	Percent
Age		
15–24	354	43.0
25–34	429	52.1
≥ 35	40	4.9
Residence		
Urban	728	88.5
Rural	95	11.5
Educational status		
No formal education	173	21.0
Primary	177	21.5
Secondary and above	473	57.5
Occupation		
Employee	311	37.8
House wife	423	51.4
Farmer	89	10.8
Religion		
Orthodox	748	90.9
Muslim	73	8.9
Protestant	2	0.2
Ethnicity		
Amhara	788	95.7
Tigre	25	3.0
Agew	7	0.9
Oromo	3	0.4
Total	823	100

### Practice of essential newborn care interventions among study participants

Cord was cut mostly by the use of reused but boiled razorblade or scissor (87%)**.** To keep the babies warm, 90.5% were immediately wrapped. Early bathing was common, with 14.6% of the babies bathed during the first 24 h after birth. Although prelacteal feeding was low 2.2% of babies were given butter as first feed (Table 2).

**Table 2:** Level of newborn care interventions during delivery and neonatal period study participants in public health facilities of Bahir Dar City administration (*N* = 823), October 2015 to August 2016

Characteristics	Frequency	Percent
Place of delivery		
Health facility	718	87.2
Home	105	12.8
Type of instrument used to cut the cord		
Reused but boiled blade or scissor	716	87.0
New blade or scissor	107	13.0
What was used to tie the cord		
Plastic band	593	72.1
Sterile thread	123	14.9
Cloth strip	107	13.0
Substance applied on the cord		
None	819	99.5
Drug	3	0.4
powder	1	0.1
How long after birth was the baby wrapped		
Immediately	745	90.5
Within 5 min	44	5.3
6–10 min	30	3.6
11–20 min	3	0.4
21–30 min	1	0.1
How long after birth was the baby first bathed		
Within one hour	3	0.4
2–6 h	19	2.3
7–12 h	50	6.1
13–24 h	48	5.8
≥ 24 h	703	85.4
How long after birth was the baby first breast fed		
Within one hour	725	88.1
2–6 h	82	10.0
7–12 h	15	1.8
13–24 h	1	0.1
≥ 24 h	0	0.0
What was given for the baby as the first feeding		
Colostrum	805	97.8
Butter	18	2.2

### ANC quality and essential newborn care practices

Among 823 pregnant women who completed the follow up time (first ANC visit to 6 weeks after birth), 227 (27.6%) received acceptable antenatal care quality during their pregnancy where as 596(72.4%) received unacceptable quality of antenatal care.

About 87.2% of the respondents delivered in the health facility (hospital or health center) but the rest 12.8% gave birth at home. All mothers who gave birth at home had no skilled birth attendant and they practiced poor essential newborn care practice.

In general the composite essential newborn care practice indices were 13.7%, with 95%CI (11.3%, 16.2%) versus 86.3%, with 95%CI (83.8%, 88.7%) for good and poor essential new born care practices respectively. Of those who received acceptable ANC quality and unacceptable antenatal care quality 24.7% and 9.6% of them had good essential newborn care practice respectively (X^2^ = 31.668, *p* < 0.000).

The composite newborn care indices of clean cord care; good neonatal feeding and optimal thermal care were 14.8% with 95%CI (12.4%, 17.3%), 87.8% with 95%CI (85.7%, 89.8%) and 85.2% with 95%CI (82.7%, 87.6%) respectively (Table 3).

**Table 3:** Practice of essential newborn care interventions across socio demographic groups of study participants in public health facilities of Bahir Dar City administration (*N* = 823), October 2015 to August 2016

Variables	Essential newborn care (ENC) practices		Frequency
	Good ENC	Poor ENC	
Age			
15–24	67(18.9)	287(81.1)	354 (100.0)
25–34	43(10.0)	386(90.0)	429 (100.0)
≥ 35	3(7.5)	37(92.5)	40 (100.0)
Residence			
Urban	108(14.8)	620(85.2)	728 (100.0)
Rural	5(5.3)	90(94.7)	95 (100.0)
Educational status			
No formal education	28(16.2)	145(83.1)	173 (100.0)
Primary	30(16.9)	147(83.1)	177 (100.0)
Secondary	55(11.6)	418(88.4)	96 (100.0)
Occupation			
Farmer	64(15.1)	359(84.9)	423 (100.0)
House wife	5(5.6)	84(94.4)	89 (100.0)
Employee*	44(14.1)	267(85.9)	311(100.0)
Religion			
Orthodox	108(14.4)	640(85.6)	748 (100.0)
Muslim	5(6.8)	68(93.2)	73 (100.0)
Protestant	0(0.0)	2(100.0)	2 (100.0)
Marital status			
Married	111(13.7)	700(86.3)	811(100.0)
Divorced	2(20.0)	8(80.0)	10 (100.0)
Widowed	0(0.0)	2(100.0)	2 (100.0)
Ethnicity			
Amhara	109(13.8)	679(86.2)	788(100.0)
Tigre	3(12.0)	22(88.0)	25 (100.0)
Awi	1(14.3)	6(85.7)	7 (100.0)
Oromo	0(0.0)	3(100.0)	3(100.0)
Grand total	113(13.7)	710(86.3)	823 (100.0)

### Predictors of essential newborn care practices

In a Generalized Estimating Equation binary logistic regression analysis, good essential newborn care practice had a positive association with antenatal care quality, use of postnatal care, being urban residence and being an employee. However parity had a negative association with good essential newborn care practice. The other socio demographic backgrounds of the study women (educational status, ethnicity, marital status and religion) were not associated with essential newborn care practice.

In multivariable Generalized Estimating Equation logistic regression analysis antenatal care quality, PNC utilization, parity and age of the mother remained to have statistically significant association with essential newborn care practices.

The odds of good essential newborn care practice among women who received acceptable ANC quality during their pregnancy was 2.31 time higher compared to those who received unacceptable quality of ANC during their pregnancy (AOR = 2.31, 95% CI = 1.47,3.65). The odds of good essential newborn care practice among women who had PNC service within 6 weeks after birth was 1.69 times higher compared to those women who had no PNC visit (AOR = 1.69, 95% CI = 1.03,2.79). The odds of good essential newborn care practice among prima-para women was reduced by57% compared to those women who were multi para or grand multi para (AOR = 0.43, 95% CI = 0.27,0.69). The odds of good essential newborn care practice among young women with age 15–24 years was 3.94 times higher compared to elderly women with age ≥ 35 years (AOR = 3.94, 95% CI = 1.12,13.91) (Table 4).

**Table 4:** Multivariable GEE logistic regression results to assess the association between ANC quality and Essential newborn care (ENC) practices (*N* = 823), October 2015 to August 2016

Variables		ENC practice		COR(95%CI)	AOR(95%CI)
		Good ENC n (%)	Poor ENC n (%)		
ANC quality	acceptable	56(24.7)	171(75.1)	3.01(2.06,4.65)	2.31(1.47,3.65)*
	unacceptable	57(9.6)	539(90.4)	1.00	1.00
Residence	Urban	108(14.8)	620(85.2)	3.14(1.25,7.90)	1.88(0.29,12.26)
	Rural	5(5.3)	90(94.7)	1.00	1.00
PNC use	Yes	39(23.4)	128(76.6)	2.34(1.55,3.69)	1.69(1.03,2.79)*
	No	74(11.3)	581(88.7)	1.00	1.00
Parity	Primi para	38(10.8)	314(89.2)	0.04(0.42,0.97)	0.43(0.27,0.69)*
	Multi/grand multi para	75(15.9)	396(84.1)	1.00	
Age of the mother (in years)	15–24	67(18.9)	287(81.1)	2.88(0.86,9.62)	3.94(1.12,13.91)*
	25-34	43(10.0)	386(90.0)	1.37(0.41,4.65)	1.37(0.39,4.82)
	≥35	3(7.5)	37(92.5)	1.00	1.00
Occupation	Employee	44(14.1)	267(85.9)	2.77(1.06,7.21)	1.65(0.24,11.46)
	House wife	64(15.1)	359(84.9)	3.00(1.17,7.67)	1.77(0.27,11.62)
	Farmer	5(5.6)	84(94.4)	1.00	1.00

*Indicates significant difference at *p* < 0.05

## Discussion

This study revealed the quality of antenatal care given for pregnant women during pregnancy had a positive effect on essential newborn care practices. This is also supported by a maternal and newborn care guide for skilled providers that noted if a woman and her family are well prepared for normal child birth as well as any possible maternal and newborn complications, the woman or baby is more likely to receive skilled and timely care needed to preserve their health and ensure survival [14]. However this finding is in contradictory to a study done Mangwi, Uganda that reported adherence to four or more ANC visits did not show statistically significant relations with newborn care practices [10]. The difference might be due to the reason that the current study tried to look the effect of antenatal care quality (contents of antenatal care services) that the women received on essential newborn care practices but the study in Uganda investigated the association between frequencies of ANC visits with the essential newborn care practices without considering the contents of services that a woman received.

The current study also noted the composite indices of good essential newborn care practices were low; only one in seven neonates had clean cord care. Unclean cord care was mainly affected by the use of any cloth strip or plastic band which was cut by unsterile scissor. This finding is also supported by a study finding in Uttar, Pradesh India that noted the number antenatal care was found to be independently associated with correct breast-feeding practices and thermal care but not with clean cord care implying more than the frequency of ANC visits; it is the quality of ANCs which matters [10, 15].

The current study finding is lower than a study done in eastern Uganda (46.9%) [16], in Mangwi, Uganda (23.7%) [10] Eastern Uganda (38%) [17]. This difference might be explained by the difference in the supplies for clean cord care at the health facilities and commitment of health professionals as well as the knowledge of her accompanies during delivery as most of newborn care practices are beyond the control of the mother alone. For example, the person who offers delivery care will cut and tie the cord without necessarily any input from the mother. In the same way drying, wrapping and bathing of the baby remains under the control of the person assisting her in the delivery or the relatives who will attend to her soon after delivery. Yet it is important to note that clean cord care is very important in preventing early neonatal infections [18].

In this study compared to clean cord care; optimal thermal care (85.2%) and good neonatal feeding interventions were well practiced (87.2).Yet about one in eight neonates had poor thermal care and neonatal feeding practices. Poor thermal care was driven by first bath before 24 h after birth. Optimal thermal care and good neonatal feeding practices were better practiced in this study compared to study done in eastern Uganda that reported 11.5% optimal feeding practices and 66.3% receiving thermal protection [16] *.*This difference might be explained by the cultural difference as breast feeding is a norm in Ethiopia even if it might not be based on WHO recommendation which is 8–12 times per 24 h. The other reason for the difference might be the background characteristics of the study participants as all of the pregnant women for the current study had four ANC visits which is also supported by a previous study in India that reported frequency of ANC visit is independently associated with breast feeding and thermal care [15]. In addition, most of the pregnant women were from urban (88.5%) and more than half of them (57.5%) had secondary and above educational status for the current study compared to less than half (48.9%) of the study women had four or more ANC visits and only 33% of them had secondary and above educational status for a study done in eastern Uganda.

The low level of composite indices of essential newborn care practices in this study was mainly driven by unclean cord care (85.2%) that might be an obstacle for the achievement of sustainable development goal of ending preventable deaths of newborns and children under 5 years of age, with all countries aiming to reduce neonatal and under-five mortality to at least as low as 12 and 25 per 1000 live births by 2030 respectively [19]. Simmilarly it might also hinder meeting the Ethiopian health sector transformation plan of reducing neonatal mortality rates to 10 per 1000 live births by 2020 [20].

In addition, post natal care utilization, mothers with young age (15-24 years) and parity had a positive association with essential newborn care practices.. . The positive association of parity with essential newborn care practice might be due to the reason that multi para or grand multi para women might have better self efficacy than primi para women in caring their child because of their previous experiences even if primi para women are very eager to care their babies with less skill.

This study finding might suffer from some limitations as the study setting did have relatively good physical access to health facilities than would be expected for most areas of Ethiopia, meaning that newborn care practices elsewhere may not be much better than those reported here. In addition, we were not able to verify the actual practices as some data were collected through recall. To minimize recall bias in this study, data collectors interview mothers about the newborn care practices while they came for immunization of their baby at 6 weeks after birth but similar studies report recall up to 1 year after birth [21, 22].

## Conclusion and recommendations

Though most mothers included in this study were good mothers (who were enrolled to their fisrt ANC visit before 16 weeks gestation, who had four ANC visits, with a better educational status and they were from urban), most neonatal interventions are not reaching newborns, indicating a “policy-to practice gap”. This implies the supplies for essential newborn care at health facilities; commitment of health professionals as well as knowledge of her accompanies during delivery are also very crucial for good essential newborn care practices as most of newborn care practices are beyond the control of the mother alone.

Good essential newborn care practice was low which was mainly affected by clean cord care though there is a statistical significant difference in the implementation of essential newborn care interventions among women who received acceptable qualityANC service and those who do not receive.

Quality of ANC service matters newborn care practices more than the frequency of visit.

To improve newborn and child survival, newborn care should be integrated into the current maternal and child health interventions, and educational program about clean cord care should be promoted both at community (involving mothers and community health workers like health extension workers) and health development armies and health facility level as part of a universal health service coverage strategy.

Distribution of low-cost pocket with essential clean and single use material (clamps and ties, single use scissors or blade) can be also promoted.

We also recommend an observational study about how cord care is done for newborns and its role to improve a good essential newborn care practice including both the individual and composite practices as the coverage of individual practices may be high and yet when combined into a composite measure might be quite low.

## Additional file

Additional file 1: **Annex I.** Participant information sheet. (DOCX 66 kb)

## Abbreviations

ANC: Antenatal Care
CI: Confidence Interval
ENC: Essential Newborn Care
FANC: Focused Antenatal Care
GEE: Generalized Estimating Equation
PNC: Postnatal Care
SPSS: Statistical Packages for Social Sciences
SSA: Sub-Saharan Africa
