# Supplementary material for: Does antenatal care service quality influence essential newborn care (ENC) practices? In Bahir Dar City Administration, North West Ethiopia: a prospective follow up study
Source: Ital J Pediatr. 2018 Aug 29;44:105. doi: 10.1186/s13052-018-0544-3 (PMC6116447; doi:10.1186/s13052-018-0544-3)
Supplement: Supplementary file 1 — Annex I. Participant information sheet. (DOCX 66 kb) [file 13052_2018_544_MOESM1_ESM.docx]

12.1. Annex I: Participant information sheet

**Title of the project**: “**Effect of Antenatal Care Service quality on the continuum of maternal and newborn heath care services**”

Principal Investigator: Tadese Ejigu

**Supervisor**: 1. Professor Mesganaw Fantahun

2. Professor Alemayehu Worku

**Coordinating office**: Addis Ababa University, School of Public Health

**Introduction:** Care during pregnancy is important for the health of the mother and the development of the unborn baby. However the importance of the care is dependent on the quality of the antenatal care.

**Purpose:** The Objective of this research is to assess the effect of antenatal care quality on the use of maternal and newborn health care services. This research undertaking is a PhD in Public Health partial fulfillment research dissertation.

**Procedure and Participation:** The method of the research is observational longitudinal study. The expected duration of the participant’s contact with the observer will be for about six months during each of the fourth antenatal visits while she is getting the antenatal care and at 6weeks after birth. You are asked to participate in this research because the trustful information which you will provide is important for the understanding of the proposed subject matter. Moreover, your particular participation is affirmed by the procedure of systematic sampling technique among all first visit pregnant mothers coming to the health facilities in the study area. While you are getting the antenatal care service the data collector will observe the service provision but the data collector will not have any involvement in the service provision. In addition to the observation you will be asked about your socio-demography, satisfaction, essential newborn care practice and post partum family planning use

**Confidentiality:** to establish secured safeguards of the confidentiality of research data, the PI will use codes during data collection period instead of using names. No person shall access the data except the PI and the supervisor. The use of information for any purpose other than that to which participants consented is unethical to the participants. The information you provide is not disclosed in the way it identified your personal characteristics and privacy. After the research defense and final work is approved by the school of public health and academic commission and university senate, the original data questionnaire will be burned in secure manner.

**Benefit:** The research does not have a short term financial, health care and capacity building benefit to the research participant as an individual or as a group but in the long run it will help the concerned organization and policy makers to have a policy consideration and direction and formulation of strategy and design of maternal and newborn health programs based on the recommendations and the findings. Moreover the research work will help as a base line data for other researches in the field.

**Risk:** The proposed research does not have any physical harm, social discrimination, psychological trauma and economic loss.

**Inducement, incentive and Compensation:** This study process has no any form of inducement, coercion and the study does not bring any risks that incur compensation.

**Results Dissemination:** The researcher is responsible for dissemination of findings moreover fully accountable to provide feedback to the health facilities under study, Woreda Health office and to the policy makers. In addition publication of the findings in peer reviewed journal will also be considered with maximum effort.

**Freedom to withdraw:** If you want to participant in the study, you have full right to with draw from the study any time you wish. This would have no effect at all on your health benefit from the health facility moreover nobody will enforce you to explain the reason of withdrawal.

**Person to Contact:** The participant has the right to ask information that is not clear about the research context and content before and or during the research work. You can contact the principal investigator and his supervisor. Moreover this research is ethically reviewed and approved by Addis Ababa University College of Health Sciences IRB. If you want more information and check about this project you can contact the following people

Addis Ababa University College of Health Sciences IRB Secretary Office Tel. 0115512876

Principal Investigator name and address: Tadese Ejigu Tel: 0920518380

**Supervisor name and address**: Professor Mesganaw Fantahun, Mobile: 0911240194,

Professor Alemayehu Worku Mobile: 0911405652.

School of Public Health, College of Health Science, Addis Ababa University, Ethiopia

**ለተሳታፊዎች የሚሰጥ መረጃ**

**የጥቱ ርዕስ፡** - « የቅድመ ወሊድ አገልግሎት ጥራት በእናቶችና ጨቅላ ህፃናት የጤና አገልግሎት አጠቃቀም ላይ ያለው ተፅዕኖ ምን ይመስላል? »

**ዋና ተመራማሪ፡-** ታደሠ እጅጉ

**ተቆጣጣሪ** ፡ 1. ፕሮፌሰር ምስጋናው ፍንታሁን

2. ፕሮፌሰር አለማዬሁ ወርቁ

**የአስተባባሪ ቢሮ፡** አዲስ አበባ ዩንቨርሲቲ የህብረሰብ ጤና ት/ቤት

**መግቢያ**፡- በእርግዝና ወቅት የሚደረግ የጤና አገልግሎት ጥራት ለእናትየዋ ጤናም ሆነ ለፅንሱ እድገት ጠቀሜታ አለው ፡፡ይሁን እንጅ ጠቀሜታው በቅድመ ወሊድ አገልግሎት ጥራቱ ይወሰናል፡፡

**ዓላማ**፡- የዚህ ጥናት ዋና ዓላማ የቅድመ ወሊድ አገልግሎት ጥራት በእናቶችና ጨቅላ ህፃናት የጤና አገልግሎት አጠቃቀም ላይ ያለው ተፅዕኖ ምን ይመስላል የሚለውን ለማወቅ ሲሆን ጥናቱ የሚደረገው የፍልስፍና ዶርትሬትዲግሪ በህብረተሰብ ጤና ለማግኘት በማሟያነት ስለሚያስፈለግ ነው፡፡

**የአካሄድና ተሳትፎ፡-** ጥናቱ የሚካሄደው ክትትል በማድረግ ነው፡፡ የጥናቱ ተሳታፊ ምልከታ/ ክትትል ከሚያደርገው ሰው ጋር ግንኙነት ሊኖረው ይቻላል ተብሎ የሚታሰበው ለ6 ወር ያህል በአራቱም የቅድመ ወሊድ ክትትሎች ወቅት አገልግሎቱ በሚሰጥበት ጊዜና ጥናቱ ተሳታፊ በወለዱ በ6ኛ ሳምንታቸው ነው፡፡ እርስዎም በዚህ ጥናት እንዲሳተፉ የተጠየቁበት ምክኒያት የሚሰጡት ትክክለኛ መረጃ ከላይ የተገለፀውን ጉዳይ ለመረዳት ስለሚረዳ ነው፡፡ በተጨማሪም የእርስዎ ተሳትፎ የሚወሰነው ከሁሉም ለመጀመሪያ ጊዜ ለቅድመ ወሊድ ክትትል ከሚመጡ እናቶች መካከል በየተሰወነ ቁጥር ርቀት በመምረጥ ነው፡፡

እርስዎ የቅድመ ወሊድ አገልግሎት በሚገኙበት ወቅት የመረጃ ሰበሳቢዋ የአገልግሎት አሰጣጡን ትከታተላለች ይሁን እንጂ መረጃ ሰብሳቢዋ በአገልግሎት አሰጣጡ ላይ ምንም አይነት ተሳትፎ አይኖራትም፡፡ከምልከታው በተጨማሪ እርስዎ ስለ ማህበራዊና ዲሞግራፊያዊ ታሪክዎን እንዲሁም በአገልግሎት አሰጣጡ ላይ ያለዎትን እርካታ፣ ለጨቅላ ህፃናት ስለሚያደርጉት የጤና እንክብካቤና ስለ ድህረ ወሊድ የቤተሰብ ምጣኔ አገልግሎት አጠቃቀም ይጠየቃሉ፡፡

**የመረጃው ምስጢራዊነት፡-** የመረጃውን ደህንነትና ምስጢራዊነት ለመጠበቅ ሲባል ዋና ተመራማሪው መረጃ ለመሰብሰብ በስም ፋንታ ኮድ ይጠቀማል፡፡ ከዋና ተመራማሪውና ተቆጣጣሪው በስተቀር ሌላ ሰው መረጃውን ሊያገኘው/ሊጠቀምበት አይችልም፡፡ መረጃውን ተሳታፊው ከፈቀደው ውጭ ለሌላ አገልግሎት ማዋል የተሳታፊውን መብት መጣስ ነው፡፡ እርስዎ የሚሰጡት መረጃ የእርስዎን ማንት በሚያሳውቅ መልኩ አይገልፅም፡፡ የምርምር ውጤቱ ለውይይት ከቀረበ በኋላና በህብረተሰብ ጤና ት/ቤት፣ በአካዳሚክ ኮሚሽንና በዩንቨርስቲው ሴኔት ከፀደቀ በኋላ መረጃው የተሰበሰበበት መጠይቅ ይቃጠላል፡፡

**ጥቅም**፡- በጥናቱ በመሳተፍዎ እንደ ግለሰብም ሆነ በቡድን በአጭር ጊዜ በገንዘብም በጤና አገልግሎት ወይም አቅምን በመገንባት የሚያስገኘው ጥቅም የለም ነገር ግን ጥናቱ ለሚመለከታቸው አካት እና ለፕሊሲ አውጭዎች የእናቶችና ጨቅላ ህፃናትን ጤና ፕሮግራም የጥናቱ ውጤት በሚሰጠው አቅጣጫ መሰረት ለመቅርፅ ይጠቅማል፡፡ በተጨማሪም መረጃው በመስኩ ለሚደረጉ ሌሎች ጥናቶች እንደመነሻ ሆኖ ያገለግላል፡፡

**አደጋ፡**- ይህ ጥናት ምን አይነት አካላዊ ጉዳት ማህበራዊ መገለል፣ የስነ - ልቦና ጉዳት ወይም ኢኮኖሚያዊ ጉድለት አያስከትልም፡፡

**መገፋፋት፣ ጥቅማጥቅምና ካሳ፡-** ይህ ጥናት የጥናቱ ተሳታፊ እንዲሆኑ ምንም ዓይነት ግፊትእንዲሁም ማሰገደድና ካሳ የለውም፡፡

**የውጤቱ ስርጭት፡-** ተመራማሪው መረጃውን የማሳወቅ ግዴታ አለበት፡፡ በተጨማሪም ጥናቱ ለተካሄደባቸው ጤና ተቋማት ፣ለወረዳ ጤና ጥበቃ ጽ/ቤት እና ለፓሊሲ አውጭዎች ግብረ- መልስ መስጠት ይጠበቅበታል፡፡

ከዚህም በተጨማሪ የጥናቱ ውጤት በታወቁ ጆርናሎች ለማሳተም የሚቻለውን ሁሉ ጥረት ይደረጋል፡፡

**የማቁረጥ መብት፡-** የጥናቱ ተሳታፊ ላለመሆንና ማቋረጥ ከፈለጉ በማንኛውም ጊዜ ለማቋጥ ይችላሉ፡፡ ይህ ደግሞ እርስዎ ከጤና ተቋሙ በሚያገኙት አገልግሎት ላይ የሚያመጣው ምንም አይነት ችግር የለም፡፡ በተጨማሪም ያቋረጡበትን ምክኒያት እንዲገልፁ ማንም አያስገድዶትም፡፡

**መጠየቅ የሚችሉት ሰው፡-** ተሳታፊዎች ማንኛውም ግልጽ ያልሆነላቸውን የጥናቱን ይዘቶች ከጥናቱ በፊትም ሆነ በጥናቱ ወቅት የመጠየቅ መብት አላቸው፡፡ ዋና ተመራማሪውን ወይም ተቆጣጣሪውን መጠየቅ ይችላሉ፡፡ በተጨማሪም ይህ ጥናት በአዲስ አበባ ዩንቨርስቲ ታይቶ ፀድቋል፡፡

ተጨማሪ መረጃ ከፈለጉ የሚከተሉትን ሰዎች ማግኘት ይችላሉ፡፡

አዲስ አበባ ዩንቨርስቲ የጤና ሳይንስ ኮሌጅ ተቋማዊ የጥናትና ምርምር የስነ- ምግባር ቦርድ የፀኃፊ ቢሮ ስልክ ፡- 0115 55 12 876

የዋና ተመራማሪ ስምና አድርሻ፡- ታደሰ እጅጉ ስልክ ፡ 09 20 51 83 80

የተቆጣጣሪ ስምና አድራሻ 1. ፕሮፌሰር ምስጋናው ፈንታሁን ስልክ፡09 11 24 01 94

2. ፕሮፌሰር አለማዬሁ ወርቁ ስልክ ፡ 09 11 40 56 52

**12.2:** Informed consent form

**Title of the project**: “**Effect of Antenatal Care Service quality on the continuum of maternal and newborn heath care services**”

I have been well aware of that this research undertaking is a PhD in Public Health partial fulfillment research dissertation which is fully supported and coordinated by AAU School of Public Health and the designate principal investigator is Tadese Ejigu. I have been fully informed in the language I understand about the research project objective that is to assess the effect of antenatal care quality on the use of maternal and newborn health care services.

I have been informed that all the information I shall provide to the interviewer will be kept confidential. I understood that the research has no any risk and no compensation. I also knew that I have the right to withhold information, skip questions to answer or to withdraw from the study any time. I have acquainted nobody will impose me to explain the reason of withdrawal. It is also enlighten there would have no effect at all in my health benefit that I get from the health facility.

I have assured that the right to ask information that is not clear about the research before and or during the research work and to contact

Addis Ababa University College of Health Sciences IRB Secretary Office Tel. 0115512876

**Principal Investigator’s Name**: Tadese Ejigu Tel: 0920518380

**Supervisor’s Name and Address**: Professor Mesganaw Fantahun Tel: 0911240194;

Professor Alemayehu Worku Mobile; 0911405652

I have read this form, or it has been read to me in the language I comprehend and understood the condition stated above, therefore, I am willing and confirm my participation by signing the consent.

Name of the participant ______________________

Agreed to participate in the study: Yes /No (mark one of them for verbal consent)

Signature ______________________ (if written consent)

Name of witness signature _________________ (Data collector, supervisor, any third person)

Signature ______________________

Date _______________________

**መረጃን መሰረት ያደረገ ስምምነት ፎርም**

**የጥናቱ ርዕስ፡** - « የቅድመ ወሊድ አገልግሎት ጥራት በእናቶችና ጨቅላ ህፃናት የጤና አገልግሎት አጠቃቀም ላይ ያለው ተፅዕኖ ምን ይመስላል? »

እኔ ይህ ጥናት የፍልስፍና ዶክትሬት ዲግሪ በህብረተሰብ ጤና ለማግኘት የሚደረግ የማሟያ ጥናት እንደሆነ እንዲሁም በአዲስ አበባ ዩንቨርስቲ ድጋፍ የሚደረግለት መሆኑን እና ዋና ተመራማሪው አቶ ታደሰ እጅዱ መሆኑን በሚገባ አውቂያለሁ፡፡ ጥናቱ የሚካሄደው የቅድመ ወሊድ አገልግሎት ጥራት በእናቶችና ጨቅላ ህፃናት የጤናአገልግሎት አጠቃቀም ላይ ያለው ተፅዕኖ ምን ይመስላል የሚለውን ለማወቅ የሚደረግ ጥናት እንደሆነ ሙሉ በሙሉ ልረዳው በምችለው ቋንቋ ተነግሮኛል፡፡

እኔ ለመረጃው ሰብሳቢው የምሰጠው መረጃ ምስጢራዊነቱ የተጠበቀ እንደሆነ ተነግሮኛ፡፡ ጥናቱ ምንም አይነት አደጋ ሆነ ካሳ የለውም ፡፡ በተጨማሪም ጥያቄዎችን ያለመመለስ ፣ የመዝለል፣ እንዲሁም ተሳታፊነቴን በማንኛውም ጊዜ የቋረጥ መብት እንዳለኝ አውቂያለሁ፡፡ ማንም ሰው ያቋረጥኩበትን ምክኒያት እንድገልፅ እንደማያስገድደኝም ተነግሮኛል፡፡ በማቋረጤ ምክኒያትም ከጤና ተቋሙ በማገኘው የጤና አገልግሎት ላይ ምንም አይነት ችግር ሊከሰት እንደማይችል ተገልፆኛ፡፡

ከጥናቱ በፊትም ሆነ በጥናቱ ወቅት ግልፅ ያልሆነልኝን ማንኛውም የጥናቱ ይዘት መጠየቅ እንደምችል ቸረጋግጫለሁ፡፡

**ጥያቄዎችን ለማቅረብ ፡**

አዲስ አበባ ዩንቨርስቲ የጤና ሳይንስ ኮሌጅ ተቋማዊ የጥናትና ምርምር የስነ- ምግባር ቦርድ የፀኃፊ ቢሮ ስልክ ፡- 0115 55 12 876

የዋና ተመራማሪ ስምና አድርሻ፡- ታደሰ እጅጉ ስልክ ፡ 09 20 51 83 80

የተቆጣጣሪ ስምና አድራሻ 1. ፕሮፌሰር ምስጋናው ፈንታሁን ስልክ፡09 11 24 01 94

2. ፕሮፌሰር አለማዬሁ ወርቁ ስልክ ፡ 09 11 40 56

ይህን ፎርም እኔ አንብቤዋለሁ/በሚገባኝ ቋንቋ ተነግሮኛል፡፡ ከላይ የተገለፁትን ጉዳዮችም ተረድቻለሁ፡፡ ስለሆነም የጥናቱ ተሳታፊ መሆኔን በፊርማዬ አረጋግጣለሁ፡፡

የተሳታፊው ስም----------------------

ለመሳተፍ ተስማምቻለሁ አዎ አልተስማማሁም

ፊርማ----------------------------(ለፅሁፍ ስምምነተ)

የእማኝ ስም---------------------------------(መረጃ ሰብሳቢ፣ ተቆጣጣሪ ወይም ሌላ ሦስተኛ ወገን)

ፊርማ-----------------------

ቀን--------------------------

12.3: Questionnaire- English Version

Section 1: Socio demographic information

Q101: Age ------------------ (in years)

Q102: Residence (specify Kebele and Got)

1. Urban (Kebele-----------------and Got----------------
2. Rural (kebele------------‘Got--------------.Tel…………..

Q103: Religion

1. Orthodox
2. Muslim
3. Protestant
4. Catholic
5. Other (specify)---------------

Q104: Ethnicity

1. Amhara
2. Tigre
3. Agew
4. Oromo
5. Other (specify) ---------------

Q105: Marital status

1. Single
2. Married
3. Divorced
4. Widowed/widower

Q106: Occupation

1. Farmer
2. House wife
3. Merchant
4. Private employee
5. Gov’t employee
6. NGO employee
7. Other (specify) ---------------

Q107: Parity------------------------

Q108: Level of education

1. Cannot read and write
2. Can ead and write ( have informal education)
3. Grade 1-4
4. Grade 5-8
5. Grade 9-10
6. Grade 11-12
7. Higher level( 12+)

Antenatal Care quality observation Checklist

| ***Section 2: Introduction and History Taking*** | | | | |
| --- | --- | --- | --- | --- |
|  | 1^st^ visit | 2^nd^ visit | 3^rd^ visit | 4^th^ visit |
| Q201: Did the health worker greet the client (and others present) in a friendly and respectful manner? |  |  |  |  |
| Q202: Did the health worker introduce her/himself and title (midwife, nurse, etc.) |  |  |  |  |
| Q203: Did the health worker ask about her LMP? |  |  |  |  |
| Q204: Did the health worker ask about Prior pregnancies? |  |  |  |  |
| Q205: Number of prior pregnancies (if any)__________ | If no prior pregnancy go to Q206 | | | |
| Q205 Did the health worker or client discuss any of the following complications for prior pregnancies |  |  |  |  |
| Q205_1: Heavy bleeding during or after delivery |  |  |  |  |
| Q205_2: Anemia |  |  |  |  |
| Q205_3: High blood pressure |  |  |  |  |
| Q205_4: Convulsions |  |  |  |  |
| Q205_5: Multiple pregnancies (twins or above) |  |  |  |  |
| Q205_6: Prolonged labour |  |  |  |  |
| Q205_7: C-section |  |  |  |  |
| Q205_8: Assisted delivery (forceps, Vacuum) |  |  |  |  |
| Q205_9: Prior abortion/miscarriage (loss of pregnancy) |  |  |  |  |
| Q205_10: Prior stillbirth (baby born dead that does not breathe or cry) |  |  |  |  |
| Q206: Did the health worker ask about or the client mentions any of the following for current pregnancy? |  |  |  |  |
| Q206_1: Vaginal bleeding |  |  |  |  |
| Q206_2: Fever |  |  |  |  |
| Q206_3: Headaches or blurred vision |  |  |  |  |
| Q206_4: Swollen face or hands |  |  |  |  |
| Q206_5: Convulsions or loss of consciousness |  |  |  |  |
| Q206_6: Severe difficulty breathing |  |  |  |  |
| Q206_7: Severe abdominal pain |  |  |  |  |
| Q206_8: Foul smelling discharge |  |  |  |  |
| Q206_9: Frequent or painful urination |  |  |  |  |
| Q206_10: Whether the client has felt a decrease or stop in fetal movement |  |  |  |  |
| ***End of Section two*** | | | | |
| ***Section 3: Tests and Treatments*** | | | | |
| Q301: Did the health worker wash his/her hands with soap or use alcohol hand rub prior to examination? |  |  |  |  |
| Q302: Did the health worker perform any of the following procedures? |  |  |  |  |
| Q302_1: weigh the client |  |  |  |  |
| Q302_1: Take the client’s blood pressure |  |  |  |  |
| Q302_3: Examine hands for edema |  |  |  |  |
| Q302_3: Perform or refer for urine test |  |  |  |  |
| Q302_4: Perform or refer for HCt/Hb test |  |  |  |  |
| Q302_5: Perform or refer for a syphilis/VDRL test |  |  |  |  |
| Q302_6: Check for signs of anemia |  |  |  |  |
| Q302_7: Palpate the client’s abdomen for uterine height |  |  |  |  |
| Q302_8: Listen to the client’s abdomen for fetal heartbeat |  |  |  |  |
| Q302_9: Did the health worker perform, inquire about, or refer for an HIV test? |  |  |  |  |
| Q302_10: PMTCT counseling? (at least once for HIV+) |  |  |  |  |
| Q303: Did the health worker give the client any of the following treatments? |  |  |  |  |
| Q303_1: Prescribed or gave iron or folic acid or both |  |  |  |  |
| Q303_2: Explained the purpose of iron or folic acid |  |  |  |  |
| Q303_3: Explained how to take iron or folic acid pills/syrup |  |  |  |  |
| Q303_4: Explained side effects of iron or folic acid |  |  |  |  |
| Q303_5: Prescribed or gave a tetanus toxoid (TT) injection |  |  |  |  |
| Q303_6: Explained the purpose of the TT injection |  |  |  |  |
| Q303_7: Importance of using ITN explained explicitly |  |  |  |  |
| Q303_8: Prescribed or gave deworming medication |  |  |  |  |
| Q303_9: Explained the purpose of deworming |  |  |  |  |
| Q303_10: Explained side effects of deworming medication |  |  |  |  |
| **Section 4: Counseling** | | | | |
| Q401: Did the health worker counsel the client in any of the following reasons to seek immediate medical care? |  |  |  |  |
| Q401_1: if she has vaginal bleeding |  |  |  |  |
| Q401_2: If she has convulsions |  |  |  |  |
| Q401_3: If she has severe headaches with blurred vision |  |  |  |  |
| Q401_4: If she has fever and is too weak to get out of bed |  |  |  |  |
| Q401_5: If she has severe abdominal pain |  |  |  |  |
| Q401_6: If she has fast or difficult breathing |  |  |  |  |
| Q402: Did the health worker counsel the client in any of the following ways about birth preparation? |  |  |  |  |
| Q402_1: Asked the client where she will deliver |  |  |  |  |
| Q402_2: Advised the client to prepare for delivery (e.g. set aside money, arrange for emergency transportation) |  |  |  |  |
| Q402_3: Advised the client to use a skilled health worker during delivery |  |  |  |  |
| Q402_4: Discussed with client what items to have on hand at home for emergencies (e.g. sterile blade) |  |  |  |  |
| Q403: Did the health worker discuss nutrition and healthy eating during pregnancy? |  |  |  |  |
| Q404: Did the health worker discuss breastfeeding? |  |  |  |  |
| Q405: Did the health worker discuss about infant immunization? (at least once) |  |  |  |  |
| Q406: Did the health worker discuss about the importance of post partum visit? |  |  |  |  |
| Q407: Did the health worker discuss family planning for use after delivery? |  |  |  |  |
| Q408: Did the health worker counsel on when to return for next visit? |  |  |  |  |
| Q409: Did the health worker speak using easy-to-understand language for the client? |  |  |  |  |
| Q410: Did the health worker ask whether the client had any questions? |  |  |  |  |
| Q411: Did the health worker write on the client’s ANC register? (*Observer: choose DK if no card)* |  |  |  |  |

**Key**: Write: **Yes =**when procedure is done; **No=** when procedure is not done.

**DK**= when you don’t know

Section 5: Outcome attributes (client satisfaction).

Please encircle the number according to the client’s agreement in the statement (3 points each).

| No. | Questions | Coding category |
| --- | --- | --- |
| 501 | Waiting time was fair | 5.Strongly agree  4.Agree  3.Uncertain  2.Disagree  1.Strongly disagree |
| 502 | Waiting area was adequate & with seats | 5.Strongly agree  4.Agree  3.Uncertain  2.Disagree  1.Strongly disagree |
| 503 | The provider was easy to understand | 5.Strongly agree  4.Agree  3.Uncertain  2.Disagree  1.Strongly disagree |
| 504 | privacy during consultation was maintained | 5.Strongly agree  4.Agree  3.Uncertain  2.Disagree  1.Strongly disagree |
| 505 | The antenatal clinic has clean latrine & adequate water supply | 5.Strongly agree  4.Agree  3.Uncertain  2.Disagree  1.Strongly disagree |
| 506 | You feel that today you received full information about ANC. | 5.Strongly agree  4.Agree  3.Uncertain  2.Disagree  1.Strongly disagree |
| 507 | I want to give birth in this health facility. | 1.Strongly agree  2.Agree  3.Uncertain  4.Disagree  5.Strongly disagree |
| 508 | you recommend your relatives &others to attend their antenatal visit in this facility | 5.Strongly agree  4.Agree  3.Uncertain  2.Disagree  1.Strongly disagree |

**Section 6: Delivery, Newborn Care and Post partum family planning use**

Q601: Place of delivery

1. Health institution 2. Home

602: Type of instrument used to cut the cord

1. Un used new razor blade /scissor
2. Used (old unboiled) razor blade /scissor
3. Household knife
4. Other (specify) ---------------

Q603: What was used to tie the cord?

1. Cloth strip
2. Clean thread
3. Rubber band
4. Other (specify) ---------------

Q604: What was put on the cord?

1. Nothing
2. Medical drugs
3. Powder
4. Cow dung
5. Ash
6. Other (specify) ---------------

Q605: After how long was the baby wrapped?

1. Immediately
2. *≤*5 minutes
3. *≤*10 minutes
4. *≤*20 minutes
5. *≤*30 minutes
6. *≤*60minutes

Q606: Cloth used for wrapping the baby

1. Old washed cloth
2. Old unwashed cloth
3. New unwashed cloth
4. New washed cloth
5. I don’t know

Q607: How soon after birth the baby was breastfed?

1. Immediately
2. Less than 6 hours
3. 6 - 24 hours
4. >24 hours

Q608: How long after birth was baby first bathed?

1. Less than 1 hour
2. 2- 6 hours
3. 7 - 12 hours
4. 13 - 24 hours
5. >24 hours

Q609: what was newborn’s first feed?

1. Breast milk/colostrum
2. Breast milk from other woman
3. Tea
4. Plain water with or without sugar
5. Formula feed
6. Other (specify) ---------------

Q610: Timing of first post natal check up

1. No post natal check up
2. Within 2 days after delivery
3. Between 3days and 6 weeks
4. After 6 weeks
5. Other (specify) ---------------

Q611: Do you use any modern contraceptive method currently?

1. Yes
2. No

Q612: When did you start after birth? ------------------- (in weeks)

Q613: Type of contraceptive in use?

1. OCP
2. Depo-Provera
3. Implant
4. IUCD
5. Other (specify)

Section 7: Check list for inventory of Facility, Equipment and supplies for FANC

**Key**: put a tick mark on the space provided accordingly.

|  | Available & satisfactory (1) | Available &un- satisfactory (2) | Not available (3) |
| --- | --- | --- | --- |
| **Availability of Infrastructure** |  |  |  |
| waiting area |  |  |  |
| Generator in case of light problemxc |  |  |  |
| Private examination room |  |  |  |
| Examination couch |  |  |  |
| Sufficient light source |  |  |  |
| Toilet |  |  |  |
| Water for hand washing |  |  |  |
| Soap |  |  |  |
| \| **Equipment** \| \| --- \| |  |  |  |
| Screen |  |  |  |
| Weight scale (adult) |  |  |  |
| Sphygmomanometer |  |  |  |
| stethoscope |  |  |  |
| Fetal scope |  |  |  |
| Thermometer |  |  |  |
| **Laboratory Facility** |  |  |  |
| Hemoglobin test reagents |  |  |  |
| Urine dip stick test for glucose |  |  |  |
| Urine dip stick test for albumin |  |  |  |
| HIV test kits |  |  |  |
| Blood group |  |  |  |
| VDRL for syphilis test |  |  |  |
| Pregnancy test |  |  |  |
| Microscopic urine test |  |  |  |
| **Drugs** |  |  |  |
| Fe-sulphate |  |  |  |
| Folic acid tablet |  |  |  |
| Antimalarial tablet |  |  |  |
| Anti helminthes |  |  |  |
| TT vaccine |  |  |  |
| Magnesium Sulfate |  |  |  |
| **FANC guidelines/reference materials** |  |  |  |
| Revised ANC cards |  |  |  |
| ANC register |  |  |  |
| Standard guideline for focused ANC |  |  |  |

12.4 Annex-II. የአማርኛ ቃለ መጠይቅ ፎርም

¡õM 1፡ ማህበራዊ ና ዲሞግራፌያዊ

ØÁo lØ` 101. °ÅT@ (u¯Sƒ) -------------------------------------

ØÁo lØ` 102. S•]Á ›É[h 1. Ÿ}T(-----------kuK?) 2. ÑÖ`(---------kuK?)

------------ÔØ ----------- ÔØ

ØÁo lØ` 103 : HÃT•ƒ

1. *`„Ê¡e ¡`e+Á”
2. ሙeK=U
3. ýa}eታ’ƒ
4. "„K=¡
5. K?L (ÃÖke)------------------------

ØÁo lØ` 104. wህ?`

1. ›T^
2. ƒÓ_
3. ›Ñ¨<
4. *aV
5. K?L (ÃÖke) ------------------------

ØÁo lØ` 105. ¾Òw‰ G<’ታ

1. ¾LÑv‹ 3. ¾ðታች
2. ¾Ñv‹ 4. vKF ¾V}vƒ

ØÁo lØ` 106. Y^

1. Ñu_ 4. ¾S”Óeƒ W^}—
2. ¾ቢƒዕSu=ƒ
3. ’ÒÈ 5. S”Óc© ÁMJ’ }sU }k×]
4. ¾ÓM }k×] 6. K?L (¾Ökce)-----------------------

ØÁo lØ` 107. Ke”ƒ Ñ>²? ¨MÅªM;---------------------

ØÁo lØ` 108. ¾ƒUI`ƒ Å[Í

1. Síõ“ T”uw ¾T}‹M 5. Ÿ9-10
2. Síõ“ T”uw ¾U}‹M 6. Ÿ11-12
3. Ÿ1-4 7. Ÿ12— ¡õM uLÃ
4. Ÿ5-8

¡õM 2: **ƒ¨<¨<p “ ¾nM U`S^**

| ØÁo lØ` | ¾SËS`Á U[S^ | | 2— U`S^ | 3— U[S^ | 4— U`S^ | |
| --- | --- | --- | --- | --- | --- | --- |
| 201. vKS<Á¨< Kc?ƒÄ­U J’ K?KA‹  ›w[­ƒ K’u\ƒ W­‹ u›¡waƒ“ uÕÅ˜uƒ SMŸ< WLUታ ›p`vFM:: |  | |  |  |  | |
| 202. vKS<Á¨< S<Á¨<”“ T°[Ñ<” ›e}ª¨<sM |  | |  |  |  | |
| 203. vKS<¨< ¾¨` ›uv KSÚ[h Ñ>²? ¾S×uƒ” k” ÖÃsM |  | |  |  |  | |
| 204. vKS<Á¨< eKuòƒ `Ó´“ ¨pƒ ÖÃsM |  | |  |  |  | |
| 205. ከዚበፊትለምንያልጊዜአርግዘውያውቃሉ | እርግዝናካልነበረወደጥያቄቁጥር 206 ይሂዱ | | | | | |
| 205. vKS<Á¨< ¾uòƒ `Ó´“” u}SKŸ} eKT>Ÿ}K<ƒ G<’@ታ­‹ ÖÃsM |  |  | |  | |  |
| 205.1 u`Ó´“ / u¨K=É ¨pƒ ŸvÉ ¾ÅU SõWe |  |  | |  | |  |
| 205.2 ¾ÅU T’e |  |  | |  | |  |
| 205.3 ¾ÅUÓòƒ SÚS` |  |  | |  | |  |
| 205.4 S”kØkØ |  |  | |  | |  |
| 205.5 G<Kƒ“ Ÿ²=Á uLÃ `Ó´“ |  |  | |  | |  |
| 205.6 ›c†Ò] UØ |  |  | |  | |  |
| ***205.7*** . u*ý^c=Ä” S¨<KÉ |  |  | |  | |  |
| 205.8 uSd]Á S¨<KÉ (hŸ=¿U 'ፎ`c?ýe) |  |  | |  | |  |
| 205.9 ¨<`Í |  |  | |  | |  |
| 205.10 S<„ ¾}¨KÅ Ií” |  |  | |  | |  |

| ØÁo lØ` | ¾SËS`Á U[S^ | 2— U`S^ | 3— U[S^ | 4— U`S^ |
| --- | --- | --- | --- | --- |
| 206. K›G<’<°ርÓ´“ ¾T>Ÿ}K<ƒ”  ØÁo­‹ ÖÃnM |  |  |  |  |
| 206.1 uwMƒ ÅU SõWe |  |  |  |  |
| 206.2 ¾S<kƒ SÚS` |  |  |  |  |
| 206.3 Ÿõ}— ¾[e Uታƒ ¾›Ã”  u»ታ |  |  |  |  |
| 206.4 ¾Ï /¾òƒ SuØ |  |  |  |  |
| 206.5 T”kØkØ /[e Uƒ |  |  |  |  |
| 206.6 ¾›}’óðe ‹Ó` |  |  |  |  |
| 206.7 Ÿõ}— ¾J’ ¾JÉ ISU |  |  |  |  |
| 206.8 SØö i ÁK¨< ðdi |  |  |  |  |
| 206.9 „KA „KA Si“ƒ/i”ƒ c=g’<  ¾ISU eT@ƒ SWTƒ |  |  |  |  |
| 206.10 ¾î”c<”penc?  Sk’e/SqU |  |  |  |  |
| ***¡õM 3 U`S^“ IŸU“*** |  |  |  |  |
| **301.** ŸU`S^ uòƒ uKS<Á¨<Ì” udS<“ ¨ÃU u›M¢M ›îÉ}FM |  |  |  |  |
| **302** uKS<Á¨< ¾T>Ÿ}K<ƒ” U`S^­‹ ›Ÿ“¨<“FM |  |  |  |  |
| 302.1 ¡wÅ}F” K¡}FM |  |  |  |  |
| 302.2 ¾ÅU Óò}F” K¡}FM |  |  |  |  |
| 302.3 ÍF TuØ /›KTuÖ<” ›Ã}FM |  |  |  |  |
| 302.4 ¾g”ƒ U[S^ ›É`ÔLM |  |  |  |  |
| 302.5 ¾ÅU T’e U`S^ |  |  |  |  |
| 302.6 ¾ÅU T’e UMŸ„‹ S•`  ›KS•^†¨<” S`UbM |  |  |  |  |
| ØÁo lØ` | ¾SËS`Á U[S^ | 2— U`S^ | 3— U[S^ | 4— U`S^ |
| 302.7 ¾TIì’<” lSƒ KŸ}ªM |  |  |  |  |
| 302.8 ¾î”c<” ¾Mw Uƒ ›ÇSÖªM |  |  |  |  |
| 302.9 ¾›?‹›Ãy U[S^ ›É[ÔLM  /U`S^¨< ¨ÇKuƒ }sU M£M |  |  |  |  |
| 302.10 zÃ[c< uÅTF ¨<eØ LKvƒ  “ƒ ¨Å î”c<’ÇÃ}LKõ  eKSÉ[Ó I¡U“ S[Í cØ}FM |  |  |  |  |
| **303 vKS<Á¨< ¾T>Ÿ}K<ƒ”**  **SÉN’>„‹ cØ}FM** |  |  |  |  |
| 303.1 ¾ÅU T’e SÉG’>ƒ |  |  |  |  |
| 303.2 eK ÅU T’e SÉG’>ƒ  S¨<cÉ ÖkT@ S[Í cØ}FM |  |  |  |  |
| 303.3 ¾ÅU S’e SÉG’>~ ’Èƒ  ”ÅT>¨cÉ ›e[É}FM |  |  |  |  |
| 303.4 cKÅUT’e SÉG’>~ K=•[¨<  eKT>‰K¨< ¾Ô’Äi Ñ<Çƒ ›c[É}FM |  |  |  |  |
| 303.5 ¾S’ÒÒ qMõ SŸLŸÁ ¡ƒvƒ  cØ}FM |  |  |  |  |
| 303.6 eKS”ÒÒ qMõ Ÿƒvƒ ÖkT@  ›c[É}FM |  |  |  |  |
| 3037 ›Ôu` SÖkU cLK¨< ÖkT@  ›e[É}ªM |  |  |  |  |
| 303.8 ¾ƒLƒM SÉG’>ƒ cØ}FM |  |  |  |  |
| 303.9 ¾ƒLƒM SÉG’>ƒ S¨<cÉ eLK¨<  ÖkT@ ›c[É}FM |  |  |  |  |
| 303.10 ¾ƒLƒM SÉG’>~ eLT>•[¨<  ¾Ô’Äi Ñ<Åƒ ›e[É}FM |  |  |  |  |

**¡õM 4 ¾U¡` ›ÑMÓKAƒ**

| ØÁo lØ` | 1— U[S^ | 2—U`S^ | 3— U[S^ | 4— U`S^ |
| --- | --- | --- | --- | --- |
| **401 ¾T>Ÿ}K<ƒ ‹Óa‹ ŸÒÖTFƒ ¨ÉÁ¨<’<’ÉƒŸU S¡[ªM** |  |  |  |  |
| 401.1 uwMƒ ÅU Sõce c=ÁÒØU |  |  |  |  |
| 401.2 S’kØkØ c=ÁÒØU |  |  |  |  |
| 401.3 ¾^e Uƒ/¾›Ã” w» c=ÁÒØU |  |  |  |  |
| 401.4 ¾S<kƒ SÚS`“ SÅŸŸU  eÁÒØU |  |  |  |  |
| 401.5 Ÿõ}— ¾J’ ¾JÉ ISU c=ÁÒØU |  |  |  |  |
| 401.6 ¾›}’óðe ‹Ó` c=ÁÒØU |  |  |  |  |
| 402 vKS<Á¨< eKT>Ÿ}K<ƒ ¾¨K=É  ´ÓÏƒ SŸbM |  |  |  |  |
| 402.1 ¾ƒ S¨<KÉ ”ÇKvƒ |  |  |  |  |
| 402.2 u¨K=É ¨pƒ eKT>ÁeðMÑ< ’Ña‹  ( KUdK? Ñ”²w 'SÕÕ¹ ¨²}) |  |  |  |  |
| 402.3 ¾T>ÁªMÉ uKS<Á” u}SKŸ} |  |  |  |  |
| 402.4 uu?}F ¨<eØ S•` eLKv†¨<  ¾TªKÍ SX]Á­‹ u}SKŸ} (KUdK?  ”îI“¨< ¾}Öuk UKß Õ”ƒ ¨²}) |  |  |  |  |
| 403 eK ›SÒÑw e[¯ƒ |  |  |  |  |
| 404 eK Ö<ƒ SØvƒ |  |  |  |  |
| 405 eK ¡ƒvƒ |  |  |  |  |
| 406 eK ÉI[ ¨K=É ¡ƒƒM |  |  |  |  |
| 407 eK ÉI[ ¨K=É ¾u?}cw U×’@  ›ÑMÓKAƒ |  |  |  |  |
| 408 eK T>kØK¨< ¾kÖa k” |  |  |  |  |
| 409 vK S<Á KTS"` ¾T>ÖkUuƒ  s”s kLM“ ÓKî ’¨< |  |  |  |  |
| 410 vKS<Á¨< c?ƒÄª ÓMî ÁMJ’Lƒ”  ØÁo "K ”ÉƒÖÃp ÁÅ`ÒM |  |  |  |  |
| 411 vKS<Á¨< upÅS ¨K=É ¡ƒƒM Ÿ`Æ LÃ  ÃS²ÓvM |  |  |  |  |

**¡õM 5:** }ÖnT>­‹ u}KÁ¿ ›ÑMÓKA„‹ LÃ ÁL†¨<” `" u}SKŸ}::

Ÿ²=I u‹ uc”Ö[»¨<eØ¾}kSÖ<ƒ’Øx‹ }ÖnT>­‹ u›ÓMÓKA~ LÃÁL†¨<”¾}KÁ¿¾`"Å[Í­‹ÁXÁK<:: }ÖnT>­‹ uT>k`wL†¨< Hdw u×U ¾T>eTS<ŸJ’“u×U eTTKG<”' u}¨c’Å[Í¾T>eTS< ŸJ’ “eTTKG<” uSeTTƒ“ vKSeTTƒ S"ŸM ŸJ’<“}Gpx” ' u}¨c’Å[Í¾TÃeTS<ŸJ’“›MeTTU” ' u×U ¾TÃeTS<ŸJ’“u×U ›MeTTU”¾T>K<ƒ”¾SMe ›T^ß¢Ê‹Á¡wu<::

| **}.l** | **ØÁo** | **¾SMe ›T^ß“ SKÁ ¢É** | **Ã²KM** |
| --- | --- | --- | --- |
| 501 | ŸÅ[eŸ<uƒ ›ÑMÓKAƒ eŸTÑ–uƒÁK¨<¾qÃÑ>²? um ’¨<:: | 5. u×U eTTKG<  4.eTTKG<  3. }Gpx  2. ›MeTTU  1. u×U ›MeTTU |  |
| 502 | ¾SqÁ x¨< um“ U‡ SkSÝ›K¨<:: | 5. u×U eTTKG<  4.eTTKG<  3. }Gpx  2. ›MeTTU  1. u×U ›MeTTU |  |
| 503 | ¾pÉS ¨K=É ¡ƒƒM ›ÑMÓKAƒ cß¨<” vKS<Á ukLK< S[Çƒ Ã‰LM:: | 5. u×U eTTKG<  4.eTTKG<  3. }Gpx  2. ›MeTTU  1. u×U ›MeTTU |  |
| 504 | ŸvKS<Á¨< Ò` ¾’u[¨< ¨<ÃÃƒ Kw‰ ’u`:: | 5. u×U eTTKG<  4.eTTKG<  3. }Gpx  2. ›MeTTU  1. u×U ›MeTTU |  |
| 505 | ¾pÉS ¨K=É ¡ƒƒM ›ÑMÓKAƒ SeÝ ¡K=’>Ÿ< ”ì<I i”ƒ u?ƒ“ um ¾¨<H ›p`xƒ ›K¨<:: | 5. u×U eTTKG<  4.eTTKG<  3. }Gpx  2. ›MeTTU  1. u×U ›MeTTU |  |
| 506 | eK pÉS ¨K=É ¡ƒƒM ¾}cÖ˜ S[Í um ’¨<:: | 5. u×U eTTKG<  4.eTTKG<  3. }Gpx  2. ›MeTTU  1. u×U ›MeTTU |  |
| 507 | መዉለድ የምፈልገዉ ከ²=I Ö?“ }sU ’ው | 5. u×U eTTKG<  4.eTTKG<  3. }Gpx  2. ›MeTTU  1. u×U ›MeTTU |  |
| 508 | K²SÊŠ“ KK?KA‹ Ÿ²=I Ö?“ }sU pÉS ¨K=É ¡ƒƒM ”Ç=ÁÅ`Ñ<S¡^KG<:: | 5. u×U eTTKG<  4.eTTKG<  3. }Gpx  2. ›MeTTU  1. u×U ›MeTTU |  |

**¡õM 6: ¨KÉ ' ¾ÚpL Ií“ƒ ¾T>Å[Ó Ø”no “ ¾ÉI[ ¨K=É ¾u?}cw U×’@ ›ÑMÓKAƒ**

ØÁo lØ` 601 ¾¨KÆuƒ x

1. Ö?“ }sU
2. u?ƒ

ØÁo lØ` 602 ƒwƒ ¾}q[Öuƒ SX]Á

1. ›Ç=e ULß/Ske
2. ØpU LÃ ¾ªK ÁM}kkK ULß/Ske
3. u=L
4. K?L (¾Öke) -----------------------

ØÁo lØ` 603 ƒw~” KTc` ¾}ÖkS<ƒ U” ’u`

1. Ú`p l[ß
2. ”îI“¨< ¾}Öuk ¡`
3. ¾ýLc+¡ v”É
4. K?L (ÃÖke)-----------------

ØÁo lØ` 604 ƒw~ LÃ ¾}kSÖ¨</¾}ÚS[¨< U” ’u`;

1. U”U 4. ¾Ÿwƒ uƒ
2. SÉG’>ƒ 5. ›SÉ
3. Æoƒ 6. K?L (ÃÖke)

ØÁo lØ` 605. Ií’< Ÿ}¨KÅ ŸU” ÁIM Ñ>²? uL ’u` ¾}ÖkKK¨<;

1. ¨Ç=Á¨<’
2. u5 Åmn ¨<eØ
3. u10 Åmn ¨<eØ
4. u20 Åmn ¨<eØ
5. u60 Åmn ¨<eØ

ØÁo lØ`606. ¾}ÖkKKuƒ Ú`p U” ›Ã’ƒ ’u`

1. ›aÑ@ ¾Öu 4. ›Ç=e ¾MታÖu

2. ›aÑ@ ¾MታÖu 5. ›L¨<pU

3. ›Ç=e ÁM}Öu

ØÁo lØ` 607. Ÿ}¨KÅ ŸU” ÁIM Ñ>²? uL ’u` Ií’< Ö<ƒ ¾Öv¨;

1. u›”É c¯ƒ
2. Ÿ2-6 c¯ƒ
3. Ÿ7-12 c¯ƒ
4. Ÿ13-24 c¯ƒ
5. Ÿ24c¯ƒ uL

ØÁo lØ` 608 Ií’< Ÿ}¨KÅ ŸU” ÁIM Ñ>²? uL ’u` ¾Öu¨<;

1. u›’É c¯ƒ ¨<eØ 4. Ÿ13-24 c¯ƒ
2. Ÿ2-6 c¯ƒ 5. Ÿ24c¯ƒ uL
3. Ÿ7-12 c¯ƒ

ØÁo lØ`609. Ií’< KSËS]Á Ñ>²? ¾}cÖ¨< UÓw U” ’u`;

1. ¾Ö<ƒ ¨}ƒ /”Ñ` 4. ¾vF”v ¨<Hue £`
2. ¾K?L W¨< ¾Ö<ƒ ¨}ƒ 5. ¾iÑ< UÓx‹
3. hÃ 6. K?L (ÃÖke) --------------------

ØÁo lØ`610. ¾ÉI[ ¨K=É ¡ƒƒM ¾ËS`g¨< SŠ ’u`;

1. ›MËS`G<U 4. Ÿ6 XU”ƒ uL
2. u 2 k” ¨<cØ 5. K?L (ÃÖke)--------------------------
3. 3k” 6 WU”ƒ

ØÁo lØ` 611. Ÿ¨K=É uL ¾u?}cw U×’@ ›ÑMÓKAƒ }ÖnT> ’­ƒ;

1. ›­ 2. ›ÃÅKG<U

ØÁo lØ` 612 KØÁo lØ` 511 ›­ ŸJ’ Ÿ¨KÆ uL ¾¨K=É Sq×Ö]Á SÖkU

¾ËS`i¨< Ÿe”ƒ XU”ƒ uL ’¨< -------------------------------------

ØÁo lØ`613 ¾UƒÖkT>¨< ¾¨K=É Sq×Ö]Á ›Ã’ƒ U”É” ’¨<;

1. ¾T>ªØ ’¡wM
2. uS`ô ¾T>cÖ¨<
3. u¡”Å e` ¾T>ku`
4. uTIì” ¨<eØ ¾T>kSØ
5. K?L (ÃÖke)-------------------------------------

**¡õM 7: የቅድመ ወሊድ አገልግሎት ለማቅረብ የሚያስፈልጉ ግባቶችን በተመለከተ ለሚደረገው ቆጠራ የማጣሪያ ዝርዝር**

| ጥ.ቁ | ግባቶች | በበቂ ሁኔታ አለ | አለ ግን በቂ አይደለም | የለም |
| --- | --- | --- | --- | --- |
| 701 | ተራ መጠበቂያ ቦታ |  |  |  |
| 702 | መብራት ሲጠፋ አገልግሎት የሚሠጥ ጄኔሬተር |  |  |  |
| 703 | የቅድመ ወሊድ አገልግሎት ለመስጠት ብቻ የሚውል ራሱን የቻለ የምርመራ ክፍል |  |  |  |
| 704 | የነፍሰጡር እናቶች መመርመሪያ አልጋ |  |  |  |
| 705 | ሽንት ቤት |  |  |  |
| 706 | በክፍሉ ውስጥ የእጅ መታጠቢያ ውሃ |  |  |  |
| 707 | ሣሙና |  |  |  |
| 708 | ባለ3 እጥፋት መለኪያ መጋረጃ |  |  |  |
| 709 | የአዋቂ የክብደት መለኪያ ሚዛን |  |  |  |
| 710 | የደም ግፊት መለኪያ |  |  |  |
| 711 | ስቴቶስኮፕ |  |  |  |
| 712 | የፅንሱ የልብ ትርታ ማዳመጫ |  |  |  |
| 713 | ቴርሞሜትር |  |  |  |
|  | ላብራቴሪ ክፍልን በተመለከተ |  |  |  |
| 714 | የእርግዝና ምርመራ |  |  |  |
| 715 | የሽንት ውስጥ ያለን የስኳር መጠን ምርመራ |  |  |  |
| 716 | የሽንት ውስጥ ያለን የፕርቲን ምርመራ |  |  |  |
| 717 | የኤች አይቪ ምርመራ |  |  |  |
| 718 | የደም አይነት ምርመራ |  |  |  |
| 719 | የቂጥኝ ምርመራ |  |  |  |
| 720 | የሄምግሎቢን ምርምር |  |  |  |
| 721 | በማይክርስኮፕ የሽንት ምርመራ |  |  |  |
|  | የመድኃኒት አቅርቦት |  |  |  |
| 722 | አይረን ሰልፌት |  |  |  |
| 723 | ፎሊክ አሲድ |  |  |  |
| 724 | የፀረ- ወባ መድኃኒት |  |  |  |
| 725 | የትላትል መድኃኒት |  |  |  |
| 726 | የመንጋጋ ቆልፍ ክትባት |  |  |  |
| 727 | ማግኒዜም ሰልፌት |  |  |  |
| 728 | ቅድመ ወሊድአገልግሎት መስጫ እስታንዳርድ ጋይድ ላይን |  |  |  |
| 729 | የቅድመ ወሊድ ካርድ |  |  |  |
| 730 | የቅድመወሊድ መዝገብ |  |  |  |
